# Multiple de novo spitzoid nevi arising within a specific red tattoo ink

**DOI:** 10.1016/j.jdcr.2024.02.012

**Published:** 2024-02-29

**Authors:** David I. Latoni, Ruth K. Foreman, Kerry Lavigne, Klaus J. Busam, Hensin Tsao

**Affiliations:** a Department of Dermatology, Massachusetts General Hospital, Harvard Medical School, Boston, Massachusetts; b Tufts University School of Medicine, Boston, Massachusetts; c Department of Pathology, Massachusetts General Hospital, Harvard Medical School, Boston, Massachusetts; d Camden Dermatology & Mohs Surgery, Rockport, Maine; e Deparment of Pathology, Memorial Sloan Kettering Cancer Center, New York, New York

**Keywords:** cutaneous oncology, dermatology, melanocytic proliferation, oncology, red ink, Spitz, spitzoid, tattoo

## Introduction

Spitz nevi are rare melanocytic lesions composed of large epithelioid and/or spindle cells. They are associated with somatic alterations in *HRAS* (including 11p amplification) and 6q23, fusions involving tyrosine kinases (eg, *ROS1*, *ALK*, *NTRK1-3*, *MET*, and *RET*), and fusions/mutations in serine/threonine kinases such as *BRAF*, *MAP3K8*, and *MAP2K1*.^1^ Spitz neoplasms are more prevalent in children, though de novo Spitz nevi arising during adulthood can occur. Segmental^2^ and eruptive^3^ forms of spitzoid neoplasms have been described though reactive spitzoid proliferation in response to a known exogenous insult has not been frequently observed. We report a case of multiple (at least 15) eruptive melanocytic proliferations with spitzoid features arising exclusively within red tattoo ink. “Spitz nevi” associated with red tattoo ink have been documented in only 1 other previous case report.^4^

## Case report

A 43-year-old male without a family history of melanoma presented to the Massachusetts General Hospital Melanoma and Pigmented Lesion Center for evaluation of recently diagnosed spitzoid tumors on his right medial thigh. Within a year after obtaining tattoos containing a new type of red ink, the patient gradually developed 15 detectable lesions at the tattooed sites, which involved the right thigh and left foot. The lesions were precisely circumscribed to areas exposed to the new red ink. Before being exposed to this new red ink, he had obtained multiple other red tattoos without any noticeable reaction. Biopsies of 3 right thigh lesions by his local dermatologist showed severely atypical spitzoid melanocytic proliferation. The patient was then referred to the Massachusetts General Hospital Pigmented Lesion Center for further evaluation.

**Fig 1:**
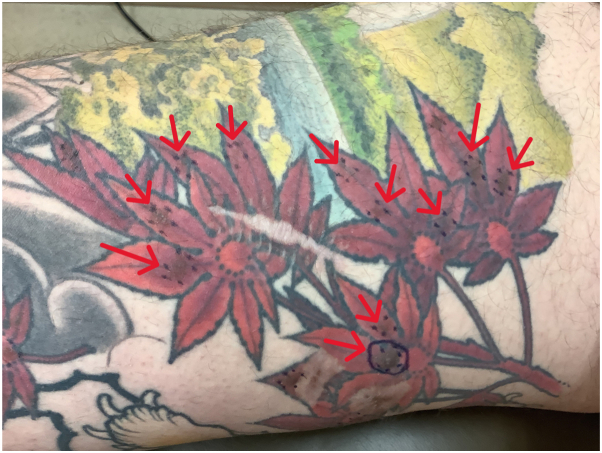
Clinical image showing 11 nevi circumscribed to the contours of *red* tattoo ink on the right thigh. Red arrows indicate the location of the nevi.

**Fig 2:**
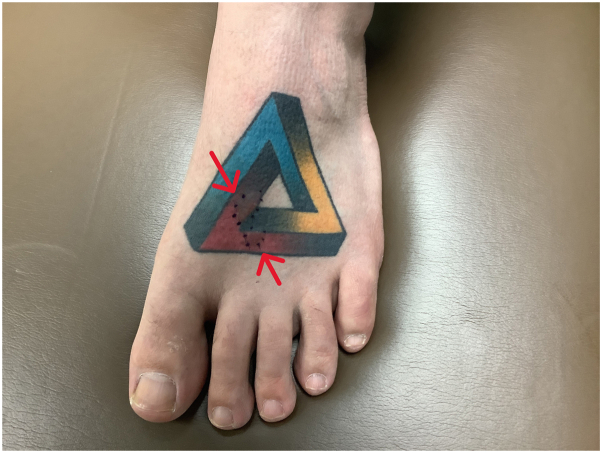
Clinical image showing 2 nevi circumscribed to the contours of *red* tattoo ink in a multicolored tattoo on the left dorsal foot (contralateral extremity). Red arrows indicate the location of the nevi.

On physical examination, there were 13 to 15 oval-shaped 0.3 to 1.5 cm firm papules throughout the right medial thigh localized only to areas with red tattoo ink (Fig 1). The contralateral left foot had 2 similarly shaped papules within another red tattoo (Fig 2) comprised of the same ink. A punch biopsy was also obtained of the left foot lesion.

**Fig 3:**
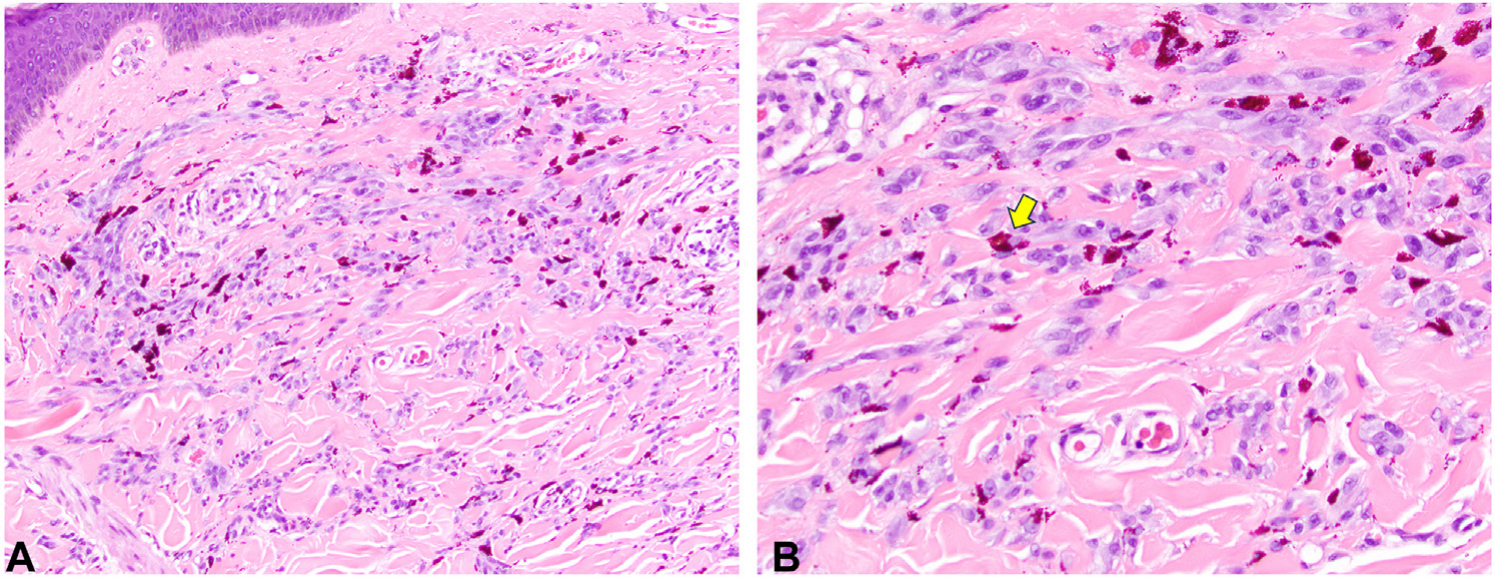
Right thigh excisional biopsy showing dermal melanocytic proliferation with spitzoid and sclerosing features. **A,** 20× and (**B**) 40× magnification. *Yellow arrow* identifies red tattoo pigment.

**Fig 4:**
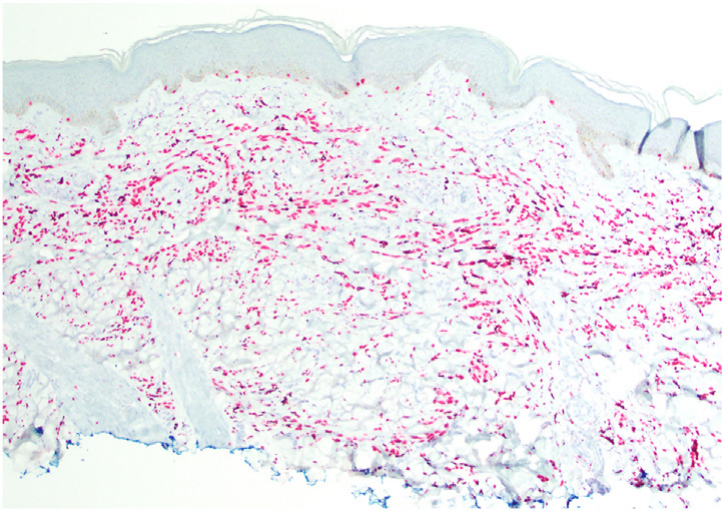
Right thigh excisional biopsy positive for SOX-10 immunohistochemical stain and negative for HMB-45, PRAME, ALK TR, and ROS1 (not shown).

Histopathological review of the prior 3 right thigh lesions showed dermal melanocytic proliferations composed of epithelioid to spindled cells and dermal sclerosis (Fig 3, *A* and *B*). The left foot lesion showed a similar dermal proliferation of ovoid melanocytes with sclerosing features. Immunohistochemistry revealed that the proliferations stained positively for SOX-10 (Fig 4), and a duplex immunostain showed weak patchy positivity for Melan-A, with a low Ki67 proliferation index. P16 expression was also retained in the melanocytic proliferations. Single nucleotide polymorphism comparative genome hybridization array analysis revealed no evidence of 11p amplification at the *HRAS* locus or obvious translocations, though the array does not comprehensively analyze for gene fusions.

## Discussion

While Spitz nevi and spitzoid tumors typically occur singly, eruptive and segmental lesions have been described. Nevares-Pomales et al reported a case of a 2-month-old female presenting with over 150 agminated nevi in a segmental distribution circumscribed to a single patch of hypopigmented skin.^2^ Histological examination of 2 lesions was characteristic of Spitz nevi. They hypothesized an underlying correlation in the pathogenesis of nevus achromicus and superimposed agminated Spitz nevi.

Ricci et al reported a case of eruptive disseminated Spitz nevi in a 12-year-old female with hundreds of lesions covering most of the skin surface.^3^ The lesions had first appeared 3 years before, with a notable dissemination in the 6 months prior to her evaluation. The authors discussed that eruptive disseminated Spitz nevi cases had a characteristic eruptive phase with many nevi appearing over a span of a few months, followed by a slower but progressive course.

We found only 1 previous report of a 28-year-old woman who presented with 2 new “intradermal desmoplastic Spitz nevi” arising within the red portion of a multicolored tattoo.^4^ The first lesions appeared 8 to 12 months after obtaining the tattoo, with the second lesion developing 3 to 4 years after. Clinical examination showed 2 adjacent 0.5-cm and 2.0-cm papules without overlying epidermal changes. Biopsy of both sites revealed desmoplastic intradermal Spitz nevi, with positive MiTF, S-100, SOX-10, and preserved p16 expression—all of which were similarly observed in our lesions.

The patient exhibited features of both agminated and eruptive nevi, with lesions bilaterally distributed and confined to areas with red tattoo ink, without typical genomic changes seen in solitary Spitz tumors. This suggests that the melanocyte proliferation might be a reaction to the red tattoo ink. As an increase in IL-18, IL-8, and IL-1α secretion has been reported in skin injected with red ink,^5^ 1 hypothesis is that the red ink incited a cytokine release, triggering melanocytic proliferation (eg, IL-1α^6^). Alternatively, these lesions could all be independent events that coincidentally occur in regions of red ink, though this possibility is statistically unlikely. Lastly, although spitzoid nevi can spread with a benign outcome, dermal metastases with strict homing to areas of red ink would also be exceedingly rare.

To date, benign and malignant melanocytic tumors are thought to be triggered by 1 or more oncogenic events. However, rare eruptive nevi challenge this mechanistic notion since the generalized process is unlikely due to coordinated cellular transformation throughout distinct pigment cells. We therefore raise the possibility that our case, along with the published report,^4^ could represent a biological reaction to a defined chemical insult. Research in this field could uncover a critical yet undefined pathway for reactive nevogenesis.

## Conflicts of interest

None disclosed.
